# Ledderhose’s Disease: An Up-to-Date Review of a Rare Non-Malignant Disorder

**DOI:** 10.3390/clinpract13050106

**Published:** 2023-09-28

**Authors:** Alexandru Tomac, Alexandru Petru Ion, Diana Roxana Opriș, Eliza Mihaela Arbănași, Claudiu Constantin Ciucanu, Bogdan Corneliu Bandici, Cătălin Mircea Coșarcă, Diana Carina Covalcic, Adrian Vasile Mureșan

**Affiliations:** 1 Clinic of Plastic Surgery, Saint Spiridon Emergency Clinical Hospital, 700111 Iasi, Romania; alex.tomac@yahoo.com; 2 George Emil Palade University of Medicine, Pharmacy, Science, and Technology of Targu Mures, 540139 Targu Mures, Romania; peti.ion@outlook.com; 3 Emergency Institute of Cardiovascular Diseases and Transplantation (IUBCVT), 540139 Targu Mures, Romania; dianaroxana.opris@yahoo.com; 4 Faculty of Pharmacy, George Emil Palade University of Medicine, Pharmacy, Science, and Technology of Targu Mures, 540139 Targu Mures, Romania; 5 Clinic of Vascular Surgery, Mures County Emergency Hospital, 540136 Targu Mures, Romania; bogdanbandici@yahoo.com (B.C.B.); catalin.cosarca@umfst.ro (C.M.C.); covalcic.carina@yahoo.com (D.C.C.); adrian.muresan@umfst.ro (A.V.M.); 6 Department of Vascular Surgery, George Emil Palade University of Medicine, Pharmacy, Science, and Technology of Targu Mures, 540139 Targu Mures, Romania

**Keywords:** Ledderhose’s disease, plantar fibromatosis, non-malignant disorders, treatments, review

## Abstract

Plantar fibromatosis (or Ledderhose’s disease) is a rare benign condition, difficult to treat, defined by gradual-growing nodules in the central medial part of the plantar fascia, with the possibility of sclerosis and shrinkage of the entire fascia or, rarely, contractures of the toes. From a histopathological point of view, it is linked to Dupuytren’s contracture of the hand and Peyronie’s disease of the penis, being part of a large group of fibromatoses, based on a proliferation of collagen and fibroblasts. Its etiology is still not fully understood, even though it has been associated with trauma, diabetes mellitus, use of anticonvulsants, frozen shoulder, alcohol consumption, and liver disease. Typically, ultrasound confirms the diagnosis, and magnetic resonance imaging is used for more aggressive and advanced types. Several conservative treatment techniques, such as steroid injections, verapamil, imatinib, radiation therapy, extracorporeal shock wave therapy, tamoxifen, sorafenib, mitomycin C, and collagenase, have been documented. When non-operative care fails, surgical measures may be considered, even though recurrence is expected. We attempted to provide a better understanding of this disease by covering all of the important aspects: its history, clinical and radiologic findings, diagnosis, pathophysiology features, conservative and surgical treatment, recurrence rate, and prognosis.

## 1. Introduction

Plantar fibromatosis (or Ledderhose’s disease) is a rare benign condition, difficult to treat, defined as a hyperproliferative disorder of the plantar aponeurosis and also by the formation of the nodules of the sole. There is evidence saying that it was first reported by Plater in 1610 [1], or that Madelung, in 1875, described the very first case [2]. Nevertheless, it is widely accepted that, after studying more than fifty cases, the German physician Dr. George Ledderhouse presented it to the world in greater detail in the year 1894 [3,4]. Including Dupuytren’s contracture of the hand and Peyronie’s disease of the penis, this condition is part of a large group of fibromatoses, based on the proliferation of collagen and fibroblasts. Even though there is no evidence that links plantar fibromatosis to a possible malignant transformation, Motolese et al. [3] reported a case of squamous cell carcinoma appearing from plantar fibromatosis.

In comparison to the palmar fibromatosis (Dupuytren’s contracture), which has been meticulously studied in the last decades, we encountered a lack of expertise when it comes to the fibromatosis of the lower extremities. In this review, we will discuss the diagnostic, treatment, operative and non-operative management, and future perspectives regarding this abnormal disorder.

## 2. Anatomy and Biomechanics of the Plantar Fascia

**Figure 1:**
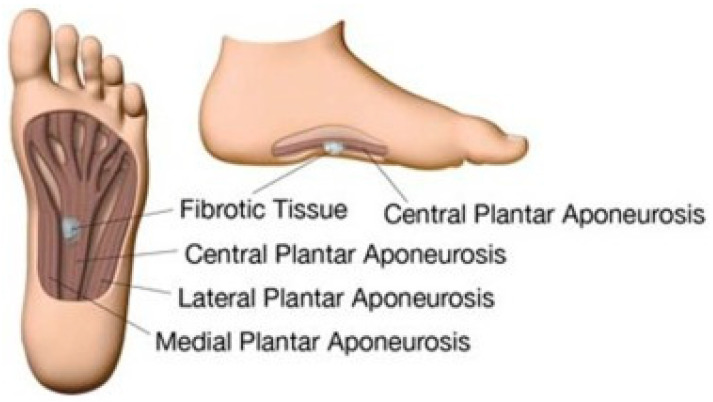
Plantar fascia with a subcutaneous nodule. Source: Reprinted from de Haan, A.; Hijmans, J.M.; van der Vegt, A.E.; van der Laan, H.P.; van Nes, J.G.H.; Werker, P.M.N.; Langendijk, J.A.; Steenbakkers, R.J.H.M. Effect of Painful Ledderhose Disease on Dynamic Plantar Foot Pressure Distribution during Walking: A Case-Control Study. *Foot Edinb. Scotl.* **2023** [6] (Elsevier Open access licensed under CC BY-NC-SA 4.0, no permission required).

The plantar aponeurosis emerges from the calcaneal tubercle and expands to the forefoot, being formed from a medial, lateral, and central part (Figure 1). The first two connect the abductor hallucis to the abductor digiti quinti pedis muscles, being referred to as the “fascia”, while the central part is much thicker and is usually considered an “aponeurosis” [5].

**Figure 2:**
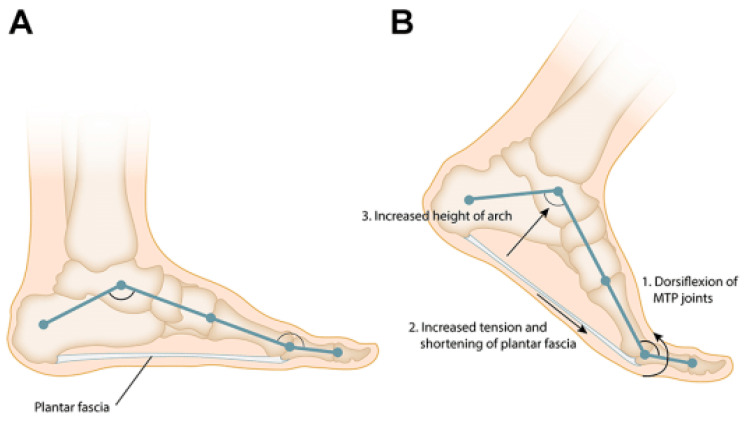
(**A**) The plantar fascia and the longitudinal arch of the foot form a truss. (**B**) Dorsiflexion of the toes during the late stance phase of gait tensions the plantar fascia around the metatarsal heads leading to an increase in the height and stability of the longitudinal arch of the foot “Windlass” mechanism. Source: Reprinted from Latt L.D.; Jaffe, D.E.; Tang, Y. Evaluation and Treatment of Chronic Plantar Fasciitis. *Foot Ankle Orthop.* **2020** [9] (SAGE Open access licensed under CC BY-NC-SA 4.0, no permission required).

It is a fibrous band that provides support to the plantar vault and has a 1–2 mm in thickness [5], having as its primary function the support of the longitudinal plantar arch [7]. Through dorsiflexion of the toes (metatarsophalangeal joints), the fascia tightens, the distance between the calcaneus and metatarsals lowers (Figure 2) and the medial longitudinal arch is uplifted [8]. When the foot is weight-bearing, the plantar aponeurosis keeps the foot arch from separating and collapsing; if it is removed, its supportive role will stop [7].

## 3. Histology

When it comes to the histopathological examination, it consists of dense fibrocellular tissue with nodular and parallel arrays of fibrocytes and fibrillar collagen, having an original cork-screw morphology. Three phases describe the evolution of this rare disease: the first one, the proliferative phase, associated with very little collagen and a large number of fibroblasts; the active one, with extra collagen and more mature fibroblasts and the third one, the maturation phase, with bigger bundles of collagen fibers [10]. There are no abnormal cells or mitotic activity and very often, all three phases can coexist together [11].

Haun et al. described the case of a plantar nodule which, from a histological point of view, was characterized by a myxoid stromal degeneration and cysts [12]. Dissimilarly to the palmar nodules, the plantar lesions come out as hypercellular [13].

## 4. Epidemiology

It affects both lower limbs in 25% of the cases [14] and it is seen more frequently in middle-aged and elderly adults, but exceptionally also in children [15], with Caucasians being the most affected age category after the age of 60 [3], with an unusual case of a nine months old baby being reported [16]. With an etiology that is still unknown [3], even though it has been linked to trauma, diabetes mellitus, use of anticonvulsants, frozen shoulder, alcohol consumption, and liver disease [17], it involves men twice more than women [18]. A hereditary susceptibility and an alteration in the structure of the collagen also need to be taken into consideration as possible incriminating factors [7].

With a prevalence of 1.75/100,000 described by Pickren et al. [19] and one of 1/100,000 by de Bree et al. [18], Ledderhose’s disease appears in the same patients with Peyronie’s disease with a frequency of 4% [20].

## 5. Clinical Presentation

Ledderhose disease is defined by gradual-growing nodules in the central medial part of the plantar fascia, with the possibility of sclerosis and shrinkage of the entire fascia or, rarely, contractures of the toes [17]. These nodules are often asymptomatic, but they can become painful when they invade the adjacent nerves, muscles, or tendons. The nodule gives no metastasis [18,21], and even though the majority of the nodules are not bigger than 3 cm in length and 2 cm in width, there have been reports of nodules up to 10.5 cm [22].

Distension and regional pressure are the first to appear in the early stage, with the late one being characterized by nodules and contractures [17]. Given the similarities with palmar fibromatosis, it is also called “Dupuytren’s disease” of the foot, appearing also in 15% of the patients with Dupuytren’s contracture, but when it comes to Ledderhose’s disease, there is no flexion of the toes as in Dupuytren [23].

Adegun et al. [24] reported the case of a 54-year-old woman with Dupuytren’s contracture of both hands and plantar fibromatosis of the right foot who presented some widespread and multiple polyp-like nodules, variable in size on the dorsal, lateral surfaces, and tip on the tongue, this being the first ever report of oral manifestation of plantar/palmar fibromatosis. There is more research needed to draw a clear line between the palmar and plantar nodules and the tongue’s polyps.

The pain that patients have to deal with in plantar fibromatosis can interfere with the ability to perform any physical activity that requires running, standing, or walking [25]. Being correlated with abnormal plantar pressure distribution, it can result in injuries, imbalance, and asymmetry in plantar pressure between the two feet, leading to a higher risk of plantar injuries [26].

De Haan et al. [6] conducted a study where it was found that in the case of patients with painful plantar fibromatosis, there is a shift of pressure towards the proximal and distal foot regions while relieving the pressure on the midfoot (the majority of the nodules are placed in the medial region of the foot). Furthermore, there is also an increase in pressure in the heel and hallux regions. Arts MLJ et al. [27] studied the difference in plantar pressure distribution between the patients with Ledderhose’s disease and a control group, finding also a decreasing mean and maximum pressure around and at the zone with the plantar nodules.

Couderc et al. [28] detailed the accelerated increase in plantar nodules in two young patients who were under treatment for spondylarthrosis with anti-tumor necrosis factor-alfa, suggesting that it could play a key role in the transformation of fibroblasts in myofibroblasts.

Vandersleyen et al. [29] reported a few cases of plantar fibromatosis evolving in patients diagnosed with metastatic melanoma who were treated with Vemurafenib, a BRAF inhibitor. It appears it leads to an activation of fibroblasts and, when combined with some genetic factors, it triggers mutations in some components of the mitogen-activated protein kinases (MAPK) pathway.

After Sammarco and Mangone Classification [30], there are four grades:

Grade 1: focal lesion with only a small area affected and no skin/muscle involved;
Grade 2: multiple areas which can extend distally/proximally, without any involvement of the skin/muscle;
Grade 3: multiple areas that can extend distally/proximally, with the involvement of skin and/or muscle;
Grade 4: multiple areas that can extend distally/proximally, with the involvement of skin and muscle.

### 5.1. Imaging

Ledderhose’s disease displays a classic pattern, and the most cost-effective, unharmful, and quick method to support the clinical diagnosis is ultrasound. It gives the chance to the examiner to correlate the ultrasound findings with the patient’s symptoms. The unaffected plantar aponeurosis is seen as a uniform band of echogenic fibers with a hypoechoic-to-anechoic background. Usually single and isoechoic, the nodule, which is about 1 cm maximum, has a heterogeneous internal structure, alongside a few thin hyperechoic septa. With no calcifications or fluid collections, the nodule follows the major axis of the plantar fascia [17].

Consistently, the color Doppler is negative, with no flow and it shows an intrinsic vascularization in only 8% of the cases [31,32]. Regularly, depending on the case series that have been analyzed, the nodules reveal posterior acoustic enhancement in 20–65% of cases, with the posterior acoustic shadowing being unusual [31,33].

**Figure 3:**
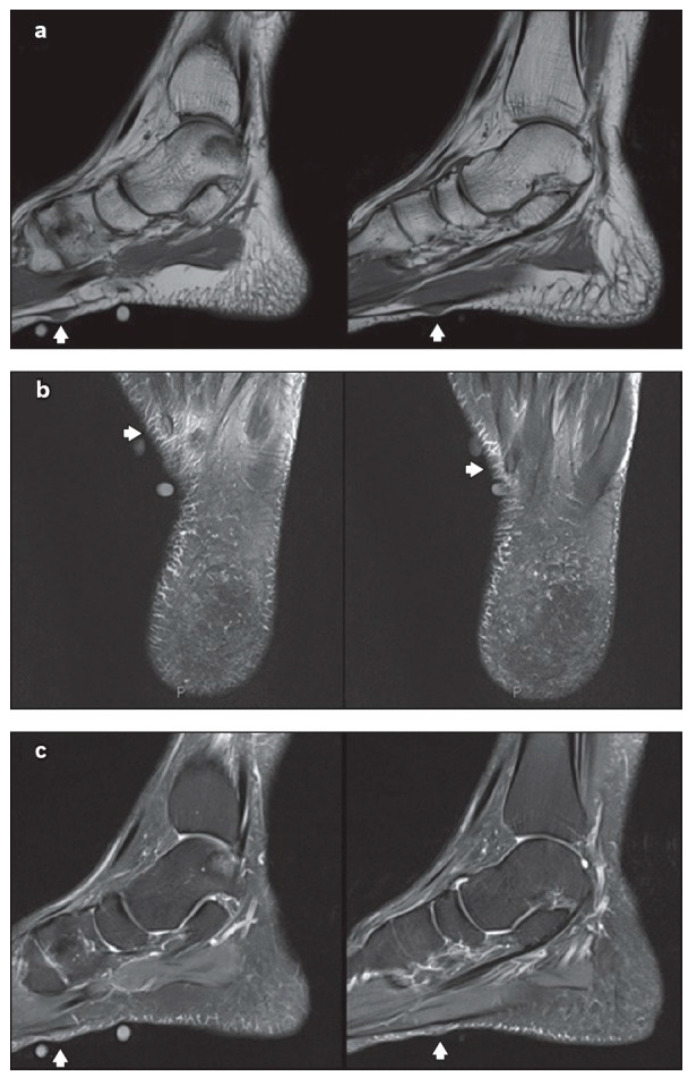
Non-contrast-enhanced magnetic resonance (MR) imaging showing two sub-centimetre nodules (arrows of **a**–**c**) along the inner band of the plantar fascia, on the left foot: (**a**) sagittal T1-W, (**b**) axial T2-W fat saturation and (**c**) sagittal proton density-weighted fat saturation MR images of the left foot. Source: Reprinted from Teo, F.; Mohamed Shah, M.T.; Wong. Clinics in diagnostic imaging. *Singapore Med. J.* **2019** [37] (Singapore Med. J. licensed under CC BY-NC-SA 4.0, no permission required).

Using MRI, the normal plantar aponeurosis appears homogeneously hypointense on both T1-weighted and T2-weighted sequences [34]. On MRI, plantar fibromatosis emerges as a nodule that has low signal intensity on T1-weighted sequences and low-to-intermediate signal intensity on T2-weighted sequences (Figure 3). The aggressive nodules are seen as hyperintense on T2-weighted sequences, because of the high cellular matrix components [35]. For better detecting the small nodules, the administration of intravenous gadolinium-based contrast could be taken into consideration, notably where the hypointense small ones are hard to differentiate from low-signal aponeurosis on non-contrast-enhanced images [34]. For the aggressive and advanced forms, the MRI is excellent for showing how deeply affected is the aponeurosis [36].

### 5.2. Differential Diagnosis

The differential diagnosis of plantar fibromatosis consists of numerous spindle cell proliferations. Nodular fasciitis, frequently met on the extremities of young adults, is a reactive proliferation of spindle cells. Low-grade fibromyxoid sarcoma appears usually in young to middle-aged adults as a profound mass concerning the trunk and the thigh, being aggressive and requiring a large excision. Neurofibromas are characterised by spindle cells with sinusoidal nuclei and an amphophilic stroma [38]. The difference between them can be determined with the aid of an MRI and/or a biopsy.

The chances of malignant soft tissue lesions such as rhabdomyosarcomas should be considered when there is a patient with an aggressive clinical presentation.

Other frequently met benign conditions associated with aponeurosis consist of plantar fasciitis, chronic aponeurotic rupture [31] and foreign body reaction [39]. In plantar fasciitis, there is usually a thickening at its calcaneal attachment, in opposition to the plantar fibromas which appear usually in the midfoot. In chronic aponeurotic ruptures, there is usually a certain history of trauma, and the tear can be diagnosed through a clinical examination or using an ultrasound. When a foreign body is presumed, ultrasound or MRI can help with the diagnosis, with foreign bodies being typically hyperechoic on ultrasound and hypointense on T1-weighted with surrounding hyperintense T2-weighted signal as a result of the granulation tissue [7].

## 6. Non-Operative Management

It is useful for most of the patients with plantar fibromatosis, with asymptomatic patients being kept under observation. If they start to have pain or to experience discomfort when walking, anti-inflammatories and physical therapy can be initiated, or they can start wearing custom-made orthotics or pads that help distribute the plantar tension differently, diminishing the stress off of the fascia [22]. It can also consist of cryotherapy, calcium channel blocker injection, and laser excision or vaporization [40].

### 6.1. Steroid Injections

Flanagan et al. [39] describe two cases of intralesional fenestration and corticosteroid injection for symptomatic plantar fibromatosis using a mixture of triamcinolone acetonide (20 mg) and mepivacaine hydrochloride (1 mL), which was deposited through an intralesional fenestration, for the first case, and without fenestration for the second one. There was a significant reduction in the size and rigidity in both of the cases.

It appears that local steroid injections lower the rate of fibroblast proliferation, intensifying the rate of apoptosis [41], with Ketchum et al. [42] stating that triamcinolone acetonide softened the keloid and hypertrophic scars, transforming the insoluble collagen into a soluble form. Pentland and Anderson [43] describe the case of a patient with recurrent bilateral multinodular plantar fibromas who 10 years ago underwent an excisional surgery on the right foot with remarkable softening of the nodules after five intralesional injections of triamcinolone acetonide. Even though intralesional corticosteroid injections appear to cut down the fibromas’ dimensions, it seems that they lack any effect on the recurrence rate [44].

### 6.2. Verapamil

It is a very well-known calcium channel blocker mainly accepted for the treatment of hypertension. Used for Peyronie’s disease, it appears it can boost the collagenase’s activity, and, at the same time, it suppresses collagen production. The transdermal and intralesional verapamil injection gel has been reported to diminish the size of fibromas by 55% to 85% [44,45], but there are no data when it comes to its effect on the recurrence rate [43], with contact dermatitis being the most frequently met adverse effect.

### 6.3. Imatinib

An amount of 400 mg of oral Imatinib was given to 40 patients over two years, without performing any surgical intervention, resulting in a 45% recurrence rate of plantar fibromas [46].

### 6.4. Radiotherapy

There are not too many studies describing the effect of radiotherapy on patients with Ledderhose’s disease. In 2003, Seegenschmiedt et al. [47] irradiated 36 feet in 24 patients, resulting in a 44% decrease in the size/number of nodules, 60% reduced pain with 11% skin dryness.

In 2015, Schuster et al. [48] used electron radiotherapy on 45 hands and 15 feet in 33 patients, resulting in 81% improved pain with strain, 70% improved pain in rest, 94% patient satisfaction, and 25% late toxicity dryness.

The most recent study, after our findings, was conducted in 2021 by de Haan et al. [49] on 102 feet on 67 patients with a median follow-up of 49 months. The results consisted of 41% complete pain response, 37% partial pain response, 0% progressive pain, and 78% patient satisfaction, with 15% skin dryness and 3% erythema.

Dryness of the skin can have a negative impact on mental and physical health in the long term [50]. It looks like the skin dryness, in plantar fibromatosis, persists over time, with it being more frequently met in patients 4 years after the treatment ended.

When it comes to the risk of radiation-induced cancer in the area that received high and low dose radiation, it is very small, at around 0.02% [47,50]. The types of cancer that have the highest possibility to appear are skin cancer or soft tissue sarcoma. Until now, from our research, there have been no cases of radiation-induced cancer after the irradiation of the sole in Ledderhose’s disease patients.

### 6.5. Extracorporeal Shock Wave Therapy

It was successfully used as treatment for chronic refractory musculoskeletal diseases, like gluteal and Achilles tendinopathies and plantar fasciitis [51,52,53,54], preceding studies showing that extracorporeal shock wave therapy (ESWT) could be implemented in distinct forms of fibromatosis, such as penile [55] and palmar fibromatosis [56] to lower the pain and to make the nodules softer, without any changes in nodule’s measurements [45,57]. When it comes to Ledderhose’s disease, a significant impact in this field was made by Frizziero et al. [58] and Knobloch et al. [59] reporting the pain-relieving effect of ESWT on six and two patients. Therefore, the first one, using two sessions of high-energy focused ESWT was able to reduce the mean visual analog scale (VAS—pain rating scale) from six to two in one week, lowering it to one after three months; the other one, applying four consecutive ESWT sessions, reduced the mean VAS from 5.6 to 0.6 at six months.

In a study performed by Hwang et al. [60], subjective pain and functional scores were considerably improved 1 week after ESWT and 34 months after it, attaining treatment success in 80% of the cases at long-term follow-up. Even though the precise mechanisms are still veiled, it is thought that the hyperstimulation of nociceptors, suppression of neurotransmitters, and increased local pain-inhibiting substances [61,62] could be responsible for the “painkiller” effect of ESWT. There is also a belief that ESWT triggers the tendon fibroblasts to synthesize extracellular matrix, which could be helpful when it comes to reducing the contracted tissue because it counterbalances the maturation process of myofibroblasts [63]. According to the study, the softening of the plantar nodules was reported in all 10 cases and the only morphologic aspect that changed on the ultrasonography was the reduced thickness. Furthermore, other than a post-treatment local soreness that healed on its own in a maximum of two days, no patient reported any adverse effects [60].

### 6.6. Tamoxifen

It is thought that anti-estrogenic drugs, like tamoxifen, diminish the fibroblast maturation, the proliferative activity of fibroblasts, and the differentiation of myofibroblasts by inhibiting the TGF-beta expression [17], but, until now, there has been no research regarding the advantages that the estrogen receptor modulating agents can bring to Ledderhose’s disease [45].

On the other hand, Okano et al. reported a case of an LGBT person who had an orchiectomy performed seven years before the intervention, who started a female hormone therapy, and whose growth of the plantar nodule started to be noticed after the estrogen therapy. This case report suggests that there may be a possibility that estrogen accelerated its growth [64].

### 6.7. Sorafenib

A study by Schoenfeld et al. [65] from 2022, conducted on five patients with Ledderhose’s disease and/or Dupuytren (four with plantar and two with both) treated with an oral multitargeted receptor tyrosine kinase inhibitor called Sorafenib, showed great results. The patients, who had been previously checked by surgeons, were inoperable due to the extensive disease or to the previous major surgeries. With an initial dose of 200–400 mg daily, the investigations reported a decrease in tumor size (by 26–64%) and tumor cellularity (by 0–62%), with no significant difference in plantar versus palmar lesions, with all the patients noticing an improvement in functionality and having reduced pain, even with a smaller dose of Sorafenib. Nonetheless, in four of them, progress in the disease’s evolution was seen when the treatment was interrupted.

### 6.8. Mitomycin C

Isolated from Streptomyces caespitosus, it is an antitumor antibiotic that has anti-fibroblastic effects according to Lee et al. [66], suppressing the growth of fibroblasts. Until now, it has been applied topically to reduce the dimensions of keloid scars and recurrent conjunctival corneal squamous cell carcinoma [67,68], with its unique side effect being self-limited and easygoing dermatitis [69,70]. Reports show that, when applied topically, it helps to avoid scar tissue development after glaucoma filtration surgery, canine subglottic surgery, and tracheal stenosis repair [71,72,73].

In the research by Amer et al. [74], after the excision of the plantar nodules, they used topical Mitomycin C (6cc, 0.4 mg/mL) on the tumor bed for five minutes. The results were completely unexpected: there was no recurrence rate of plantar nodules when topical Mitomycin C was associated with surgical resection compared to the surgical treatment alone. So, it appears that Mitomycin C inhibits the growth of these tumors by obstructing fibroblastic activity. Also, according to this study, the topical application of Mitomycin C was safe and there was no augmentation in the toxicity of the wound or any delayed wound healing.

### 6.9. Collagenase

De Vitis et al. [75] brought to our attention the case of a 59-year-old man with symptomatic, bilateral Ledderhose’s disease who was treated with intranodular injection of collagenase of Clostridium hystoliticum (CCH), with the nodule being proximal to the metatarsophalangeal joint on both sides. After six days postoperatively, the nodule was not detectable. The same procedure was repeated to the other limb and after more than one year, there was no noticeable plantar nodule and no recurrence.

There is only one other study regarding the use of Clostridium hystoliticum collagenase, conducted by Hammoudeh [76], on a patient who was already treated with a series of steroid injections and who underwent a plantar fasciectomy. Injections with CCH were administered three times, without any results; the author associated the failure with the anatomical and pathological aspects that are different from Dupuytren’s disease, but it could be a possibility that the scar tissue also played a key role.

From the experience of de Vitis et al. and their successful treatment of a patient with bilateral plantar fibromatosis, the CCH injection appears to be safe and effective, the nodule’s dimensions preventing the substance from spreading systemically. At the same time, there are no scars, and there is no need to hospitalize the patient, allowing them to return immediately to their everyday life. Also, forced extensions of the plantar fascia could be avoided as, through walking, the medicine distributes inside the nodule.

## 7. Operative Management

It is very important to look very carefully in order not to damage the arteries that sustain the vascularity of the dermis, with the fascia being left perpendicular to the skin and curved in a centrifugal way. This points out to the increased risk of ischemia and necrosis of zigzag incisions medial to the midline of the foot, while the longitudinal incisions that are made adjacent to the medial plantar arch can result in painful and hypertrophic scars because they involve the Langerhans’ tension lines of the skin. Meanwhile, incisions that involve the lateral part of the foot could cause pain and soreness [11].

When it comes to the nerves, it is crucial to complete the dissection very precisely in order not to injure the medial or the plantar nerve (terminal branches of the tibial nerve), which could potentially cause long-lasting paresthesiae [11].

Excision should be taken into consideration when the disease results in functional disability or the total flexion deformity is greater than 30 degrees [77].

Surgical treatment is branched into three categories: local, wide, and complete fasciectomy [17,31,78]. In 2016, Lu et al. [79] performed a study on 13 patients who underwent wide excisions, having a recurrence rate of 15%. In 2008, Van der Veer et al. [80] studied a group of 27 patients who underwent marginal excision, wide excision, and total plantar fasciectomy, having a recurrence rate of 25% following total plantar fasciectomy and 100% following marginal excision. An amount of 11 patients with marginal, wide excision or complete total fasciectomy were investigated by Durr et al. [81], resulting in a recurrence rate of 55% following marginal excision, 78% following wide excision, and 50% following total fasciectomy. Aluisio et al. [82], exploring 30 patients with marginal, wide excision, and subtotal plantar fasciectomy, found a recurrence rate of 75% for marginal and wide excision and 23.5% for subtotal fasciectomy, while Wapner et al. [83] performed a study on 12 patients with wide excision. While the technique consisted of delayed split-thickness skin graft closure, the recurrence rate was 20% for the primary and 28.6% for the revision procedures.

Even though the fasciectomy appears to be the most adequate treatment, there are higher possibilities of complications than the wide excision. There is a high injury risk of the first branch of the lateral plantar nerve (Baxter’s nerve) during a total fasciectomy. Pontious et al. [84] reported the case of a patient who, after the excision of a fragment of the plantar fascia, developed hammertoes because of an important deficit in digital stabilization.

**Figure 4:**
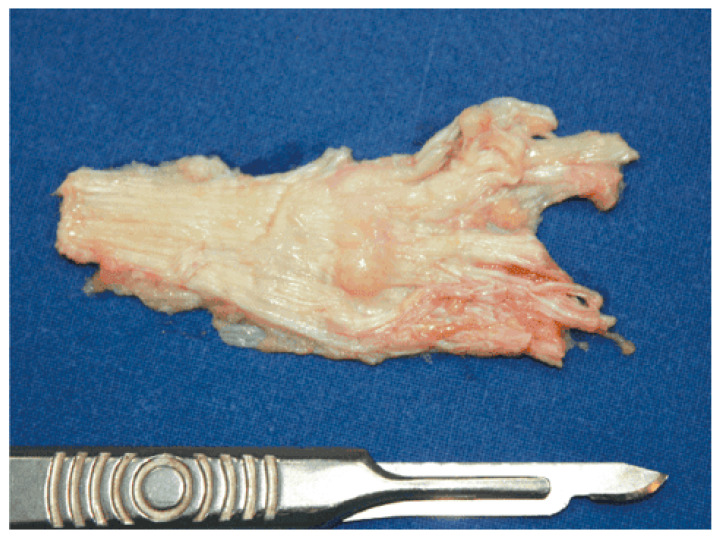
Macroscopic aspect of the modified plantar aponeurosis revealing nodules of different dimensions. Source: Reprinted from Neagu, T.P.; Tiglis, M.; Popescu, A. Clinical, histological and therapeutic modern approach of Ledderhose disease. *Rom. J. Morphol. Embryol.* **2018** [86] (Rom. J. Morphol. Embryol. Open access licensed under CC BY-NC-SA 4.0, no permission required).

**Figure 5:**
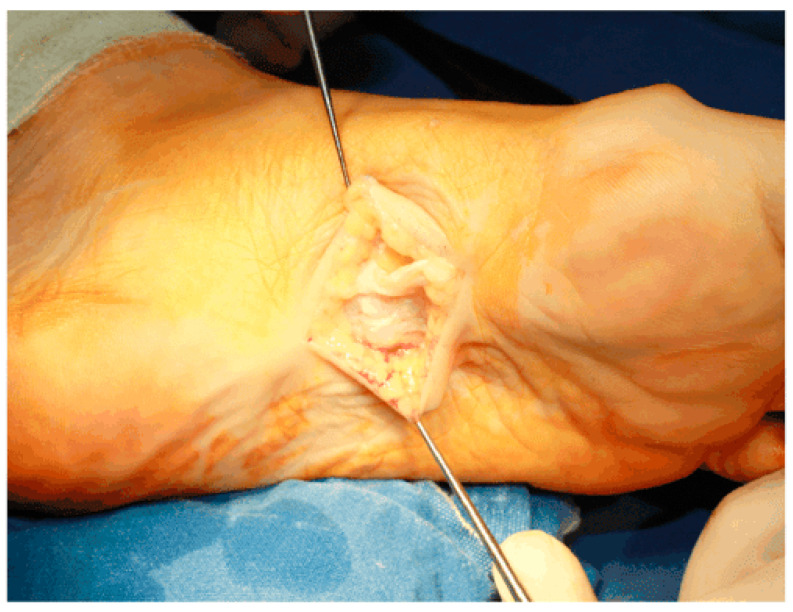
Intraoperative aspect of the nodules and plantar aponeurosis. Source: Reprinted from Neagu, T.P.; Tiglis, M.; Popescu, A. Clinical, histological and therapeutic modern approach of Ledderhose disease. *Rom. J. Morphol. Embryol.* **2018** [86] (Rom. J. Morphol. Embryol. Open access licensed under CC BY-NC-SA 4.0, no permission required).

Kan and Hovius [85] reported the case of two brothers whose surgical interventions consisted of full-thickness skin grafts with free vascularized upper lateral arm flaps. They were operated on multiple times, and after it reappeared, it was performed a large dermofasciectomy of their Dupuytren’s and Ledderhose’s diseases. With the recurrence of just one nodule of the sole, they were pain free. Figure 4 and Figure 5 show the macroscopic and intraoperative aspect of nodules [86].

### 7.1. Endoscopic Subtotal Fasciectomy of the Foot

In 2016, Lui et al. [87] developed an endoscopic technique of subtotal fasciectomy for patients who do not respond to conservative treatment, although it is contraindicated in the case of neurovascular bundles of the sole or lesions that have already invaded the plantar skin or muscles of the sole. Because the incisions are smaller than those used in open access, there is less possibility of developing hypertrophic scars. Meanwhile, by making only two small portal skin incisions, the risk of ischemia or necrosis is reduced.

### 7.2. Complications after the Surgery

Patients with a family history of plantar fibromatosis, who develop a post-operative neuroma, or those who have multiple/bilateral nodules, present a greater possibility of requiring another surgery [18].

Meanwhile, complications like painful scarring, dehiscence, or a deficit in the height of the medial longitudinal arch (if a subtotal fasciectomy is performed) can appear [88].

### 7.3. Recurrence Rate after Excision

It has been observed that the recurrence rate following excision is around 60% [80], with some findings claiming that phenobarbital, in epileptic patients, could augment the chances of recurrence [89]. According to one study, females with larger tumors have a greater probability of recurrence [74].

## 8. Conclusions

The most effective therapy for plantar fibromatosis is still being developed as numerous conventional conservative medicines and cutting-edge treatment alternatives have been researched with various degrees of success. Conservative therapies continue to be first-line alternatives for symptomatic care, given the benign nature of this illness; however, solid long-term studies supporting their usage are still lacking. In order to choose the best therapy algorithm, further research is required. Although recurrence of the nodules is not unusual, there are a number of surgical procedures available for persistent or extremely aggressive instances.

## Data Availability

Not applicable.
